# Supplementing Best Care with Specialized Rehabilitation Treatment in Parkinson’s Disease: A Retrospective Study by Different Expert Centers

**DOI:** 10.3390/jcm13102999

**Published:** 2024-05-20

**Authors:** Maria Felice Ghilardi, Angelo Quartarone, Alessandro Di Rocco, Rocco Salvatore Calabrò, Sheng Luo, Hongliang Liu, Monica Norcini, Margherita Canesi, Veronica Cian, Marianna Zarucchi, Paola Ortelli, Daniele Volpe, Leila Bakdounes, Davide Castelli, Alessio Di Fonzo, Giulia Franco, Emanuele Frattini, Laura Avanzino, Elisa Pelosin, Carla Ogliastro, Roberto Ceravolo, Giovanni Palermo, Luca Tommasini, Daniela Frosini, Lucilla Parnetti, Nicola Tambasco, Pasquale Nigro, Simone Simoni, Peter Schmidt

**Affiliations:** 1 Cellular & Biomedical Sciences Department, CUNY School of Medicine, Molecular, New York, NY 10031, USA; lice.mg79@gmail.com; 2 The Graduate Center, City University of New York, New York, NY 10016, USA; 3 IRCCS Centro Neurolesi “Bonino-Pulejo” Messina, 98124 Messina, Italy; aquartar65@gmail.com; 4 Department of Neurology, Northwell System, New York, NY 11040, USA; adirocco1@northwell.edu; 5 Department of Population Health, Duke University, Durham, NC 27708, USA; sheng.luo@duke.edu (S.L.); hongliang.liu@duke.edu (H.L.); 6 NYU Langone Health, Department of Neurology, New York, NY 10016, USA; 7 The Gravedona e Riuniti Ospedale, 22015 Gravedona, Italy; margheritag2007@yahoo.it (M.C.); cian.veronica@gmail.com (V.C.); marianna.zarucchi@gmail.com (M.Z.); ortellipaola73@gmail.com (P.O.); 8 Villa Margherita—S. Stefano Riabilitazione, 36057 Vicenza, Italy; dott.dvolpe@libero.it (D.V.); leilabak86@gmail.com (L.B.); davide.castelli1995@gmail.com (D.C.); 9 Department of Neurology, IRCSS Fondazione Ca’ Granda, Ospedale Maggiore Policlinico di Milano, 20122 Milano, Italy; alessio.difonzo@policlinico.mi.it (A.D.F.); giulia.francomed@gmail.com (G.F.); emanuele.frattini@gmail.com (E.F.); 10 Department of Neuroscience, Rehabilitation, Ophthalmology, Genetics, Maternal and Child Health, IRCSS Policlinico San Martino, 16132 Genoa, Italy; lavanzino76@gmail.com (L.A.); elisapelosin@gmail.com (E.P.); carlaogliastro@gmail.com (C.O.); 11 Department of Experimental Medicine, Section of Human Physiology (LA), University of Genoa, 16126 Genoa, Italy; 12 Department of Clinical and Experimental Medicine, Center for Neurodegenerative Diseases-Parkinson’s Disease and Movement Disorders, University of Pisa, 56126 Pisa, Italy; roberto.ceravolo@unipi.it (R.C.); palermo.giovanni85@gmail.com (G.P.); lucatommasini92@gmail.com (L.T.); danielafrosini80@gmail.com (D.F.); 13 Ospedale Santa Maria della Misericordia, 06156 Perugia, Italy; lucilla.parnetti@unipg.it (L.P.); n.tambasco@libero.it (N.T.); pasquale.nigro1987@gmail.com (P.N.); simone.simoni@unipg.it (S.S.)

**Keywords:** intensive neurorehabilitation, neurodegenerative disorders, aerobic exercise, multidisciplinary care, Parkinson’s disease

## Abstract

**Background**: This is a retrospective longitudinal study comparing 374 patients with Parkinson’s disease (PD) who were treated in centers offering a specialized program of enhanced rehabilitation therapy in addition to expert outpatient care to 387 patients with PD, who only received expert outpatient care at movement disorders centers in Italy. **Methods**: The data are from subjects recruited in the Parkinson’s Outcome Project (POP) at six Italian centers that are part of a multicenter collaboration for care quality improvement (the Fresco Network). The effects were measured with a baseline and a follow-up clinical evaluation of the Timed-Up-and-Go test (TUG), Parkinson’s Disease Questionnaire (PDQ-39), and Multidimensional Caregiver Strain Index (MCSI), the number of falls and hospitalizations for any cause. We used a generalized linear mixed model with the dependent variables being the response variable, which included the covariates demographics, evaluation, and treatment variables. **Results**: We found that the subjects who underwent specialized enhanced rehabilitation had a better motor outcome over time than those who were managed by expert neurologists but had participated in community programs for exercise and other allied health interventions. The greatest effects were seen in patients in the early stages of the disease with a high amount of vigorous exercise per week in the last six months. Similar effects were seen for PDQ39, MCSI, the number of falls, and hospitalization. **Conclusions**: Long-term benefits to motor function and the quality of life in patients with PD and burden reduction in their caregivers can be achieved through a systematic program of specialized enhanced rehabilitation interventions.

## 1. Introduction

Multidisciplinary care modalities have been linked to better outcomes in Parkinson’s disease (PD) care [1,2]. Patients with PD are at a high risk of inpatient admissions, and 58% of admissions are associated with the complications of this disease [3]. Multidisciplinary care can predict reduced hospitalization and re-hospitalization [4]. For the majority of patients managed in subspecialty movement disorders centers, referrals for multidisciplinary team care are delayed until patients experience severe disease [5]. An increasing knowledge of non-medical interventions enhances our understanding of how coordinated care across multiple disciplines can enable dramatic improvements in health-related quality of life (HRQL) [6,7]. However, health systems often fail to provide the infrastructure and training necessary to provide access to these benefits [8].

Structured models for intense and integrated multidisciplinary interventions have been developed, targeting non-medical interventions to compensatory mechanisms specific to PD patients [9], and training programs have been developed to facilitate the formation of teams to deliver them [10]. In particular, one of our protocols was demonstrated to be beneficial if it was started soon after the PD diagnosis [11]. A grant-supported collaboration of six expert movement disorder centers was formed to pursue care quality improvement (Fresco Network). This network includes centers that have sought to refine these intensive rehabilitation strategies and multidisciplinary care [12]. These approaches have the evidence of efficacy in small randomized controlled trials (RCTs); however, support for this approach would benefit from real-world evidence, comparing best practices across centers.

To address these issues at least in part, we used data from the longitudinal natural history study, the Parkinson’s Outcome Project (POP) [13]. This observational study was developed to track the outcomes of care across leading PD centers and was profiled as a useful tool for identifying and disseminating the best practice guidelines [14]. A non-blinded comparison of expert care across care models is an important supplement to a formal randomized controlled trial (RCT), because every patient receives the benefit of a carefully considered standard of care believed by clinicians to be optimal at a tertiary referral center. The analysis of real-world evidence from a clinical practice does include a risk of bias [15]. We address these risks below. However, outcomes in double-blind RCTs in PD commonly show a placebo benefit due to the expectation of therapeutic improvement [16]. Because our study focused on the efficacy of care models on clinical outcomes and the difficulty of blinding participants in this context, this unblinded study of care previously established in blinded RCTs was appropriate.

The platform study POP includes subjects recruited from the six centers in the Fresco Network: two centers that offer inpatient and outpatient intensive rehabilitation (“IR centers”, i.e., Gravedona and Riuniti Hospital, Gravedona; Villa Margherita, Vicenza), and four centers that provide traditional outpatient care (“TO centers”, i.e., IRCSS Ca’ Granda, Milano; Ospedale Santa Chiara, Pisa; IRCCS Policlinico San Martino, Genova; Ospedale Santa Maria, Perugia). To confirm the prior RCT evidence supporting the impact of the intensive rehabilitation approach in the real world, we used data previously collected in this Italian network to control for the health system, cultural, and social differences. Our team thus conducted a retrospective longitudinal study analyzing the outcomes of patients with PD receiving the expert-optimized best care model at the IR centers and those of patients receiving the expert-optimized best care model at the TO centers. Models were developed to determine the dependence of outcomes on different factors, with specific regard to the intensity and the duration of weekly exercise and other factors.

## 2. Materials and Methods

Drawing from the longitudinal POP database, subjects with data from at least two visits (average of 2.42 visit/patient) in an average period of 19.56 months were included in these analyses. We selected subjects that, at their baseline, had a diagnosis of PD with the confidence of the expert movement disorder specialist greater than 50% (as determined in the POP studies [13,14]) and Hoehn and Yahr (H–Y) stage 1 to 4. As per POP instructions [13,14], the diagnosis was based on the opinion of an expert movement disorder specialist practicing in a center of excellence for the care of PD, certified by the Parkinson’s Foundation. Subjects were then classified, first, in two groups by their recruitment “center”, either an IR center or a TO center. Second, we classified all patients in terms of “exercise” as belonging to one of three subgroups based on the intensity and the weekly duration of exercise:

*1. Intense vigorous exercise (S1).* Subjects that satisfied the following criteria: twelve hours or more of total weekly exercise with at least four hours per week of vigorous or moderate exercise. There were no specific requirements about participation in rehabilitation programs.

*2. Moderate exercise with rehabilitation (S2)*. Subjects that reported less than twelve hours but more than four hours of weekly exercise and underwent sessions of rehabilitation treatment in the prior year.

*3. Minimal or no exercise (S3)*. Subjects that reported less than four hours a week of exercise. There were no requirements for participation in rehabilitation programs.

The exercise activity was based upon the POP categorization and definition: 1. Vigorous exercise (such as jogging or running, fast biking, stair climbing for exercise, swimming laps, weight lifting); 2. moderate exercise (fast/brisk walking pace or walking in hills, dancing, tai chi, yoga, Pilates, arm or leg cycling, pool aerobics); 3. light exercise (walking at a leisurely pace, chores in the house/yard, seated exercise routine) [13,14].

To be noted, during the study period, patients of the IR centers underwent two periods of intensive rehabilitation (at an average interval of 12 months), overseen by a comprehensive care team and delivered over a two- to four-week inpatient stay for at least three hours a day. Briefly, such treatment included the following: aerobic exercises targeting cardiovascular health, balance; postural and gait exercises with or without stabilometric platforms and aquatic therapy; compensatory training with occupational therapy to improve autonomy in daily living activities; and cognitive therapy and psychosocial support. The outcome measures were selected from the POP dataset to span the domains impacted by PD and were targeted by the interventions. To measure the impact of exercise and rehabilitation interventions, changes in a direct measure of mobility, the Timed-Up and-Go test (TUG) was selected [17]. Motor impairment, and in particular balance dysfunction, was also evaluated by considering the frequency of falls in the prior three months with a novel measure previously described [13,18]. Healthcare utilization was evaluated through a proxy measure of the number of hospital admissions. The Parkinson’s Disease Questionnaire (PDQ-39) was used to evaluate health-related quality of life (HRQL) [19], and the Multidimensional Caregiver Strain Index (MCSI) provided a measure of the caregiver strain [20]. All outcomes and covariates were collected according to the POP protocol [13].

### Statistical Analyses

The differences between the two groups at the baseline were tested using unpaired *t*-tests for continuous normal distributed variables, while chi-square tests were used to compare the distribution difference of categorical and not-normally distributed variables. Models were developed to determine the dependence of outcomes on subject demographics (age, sex, time since PD diagnosis), clinical evaluation (the H–Y stage, with stages 1 and 2 combined) and treatment approach (participation in intensive rehabilitation, referrals to allied health, computed levodopa equivalent dose, or LED, and exercise subgroups) [21]. For each outcome measure, we utilized a generalized linear mixed model with the dependent variables being the response variable. The model included the demographics, evaluation, and treatment variables as covariates. Models employed the random intercept and random slope of follow-up time. We used the regression approach with skewed t distribution assumption in the R-skewed package. Statistical analysis was overseen by one of us (SL) who is an expert statistician, with deep experience in RCT and observational study analysis in PD [22,23].

## 3. Results

A total of 761 patients met the inclusion criteria, with 374 patients from the two IR centers and 387 patients from the four TO centers. The characteristics of the two groups of patients (IR vs. TO centers) at the baseline are reported in Table 1.

Briefly, at the baseline, the two groups significantly differed in terms of the H and Y stages (fewer patients in stage 1 and more in stages 3 and 4 in the IR center group compared to the TO centers), disease duration (longer in the IR center groups), motor performance (TUG better in the IR center groups), falls (fewer patients reported falls in the TO center groups), and HRQL (PDQ39 slightly better in the TO center groups). The patients with more than 90% diagnostic certainty of PD represented 79% and 82% of the data set in the IR and TO centers, respectively. The number of participants in the S1 subgroup (*Intense vigorous exercise*) was 40, 182 into the S2 (*Medium exercise with rehabilitation)*, and 539 into the S3 (*Minimal or no exercise*). S1 subjects reported a mean 18.7 h of total weekly exercise (standard deviation: 6.01 h). S2 subjects reported a mean 7.17 h of weekly exercise (SD: 3.43 h), and S3 subjects reported a mean of 1.01 h of weekly exercise (SD: 1.23 h).

We then analyzed the relationship between the exercise subgroup assignment and the progression of TUG with a skew-T generalized linear mixed model with covariates at the baseline that best fitted the data for the TUG measures, both without and with push off. The results of the two analyses in 695 patients for a total of 1467 visits are reported in Table 2 and Table 3. Briefly, both analyses showed that the subjects seen at the IR centers achieved a better mobility outcome than those seen in the TO centers. In addition, there were significant effects of the amount of exercise per week and the H and Y stage, suggesting that the greatest effects were seen with a high amount of vigorous exercise per week and in the early stages of the disease. A moderate amount of exercise, even in combination with rehabilitation sessions performed outside the IR centers, did not achieve the same benefit of highly intensive exercise.

We then applied the same approach for falls (0 = “no falls”; 1 = “one or more falls”) and the number of hospital admission. In terms of falls, there was a trend toward significance for falls being less frequent in patients recruited at the IR centers (*p* = 0.064) than at the TO centers (Table 4). Female sex, younger age, earlier disease stages, disease duration at the baseline and lower LED at the baseline were significant covariates (all: *p* < 0.05). The intensity and the amount of exercise were not associated with frequency of falls, as shown by the comparison between S1 and S2 (*p* = 0.141) and between S1 and S3 (*p* = 0.127).

Similarly, as reported in Table 5, we found a lower rate of hospital admissions among subjects seen in the IR centers (*p* = 0.001). This result was associated with shorter disease duration at the baseline (*p* = 0.024), and earlier PD stages (H and Y 1–2 vs. H and Y 3: *p* = 0.001; H and Y 1–2 vs. H and Y 4: *p* = 0.019).

As shown in Table 6, HRQL measured with PDQ39 was better over time for patients treated in the IR centers than the TO centers (*p* = 0.018). Also, female sex, younger age, earlier disease stages, lower LED, shorter disease duration, and intensive exercise were significant covariates (all *p* < 0.05).

Finally, we obtained similar results when we analyzed MCSI (see Table 7), an index of the caregiver burden, confirming that the progression and severity of the disease exact a big toll from caregivers, while the participation of patients in IR centers and intensive exercise can also yield a beneficial effect for caregivers.

## 4. Discussion

The results of this longitudinal study suggest that patients who were exposed to intensive rehabilitation programs at the IR centers achieved, on average, better motor outcomes compared to other patients that were cared for in the TO centers. Given past RCT evidence supporting the IR center approach, this comparison of expert care across sites provides further evidence for this care model. The analyses of this real-world dataset confirmed the beneficial effect of vigorous exercise, although we found that more than twelve hours of weekly exercise with at least four hours of vigorous exercise was beneficial only in patients in the early stages of the disease. These findings were reflected in both the clinical and HRQL outcomes. Finally, intensive rehabilitation and vigorous exercise of patients had a positive impact on the financial, psychological, social, and personal strain of the caregivers measured with MCSI, probably providing some respite and recovery that are essential for the caregivers’ quality of life.

The present findings are in line with previous results that showed an improvement in both motor function (including TUG evaluation) and HRQL in patients that underwent intensive rehabilitation at the IR centers [11,24,25]. Those investigations showed evidence for a decreased rate of clinical symptom progression at a two-year follow-up and improved HRQL. Building on those results, this study demonstrates that the IR approach offers benefits not seen at the TO expert centers that shared the same cultural and health system with the IR centers. In addition, the expert approach we used in our statistical model controlled for the demographics, disease stage, and treatment approaches. The data set came from the POP study that was conducted with clinical site coordination and monitoring visits, as well as with the in-person training of study personnel and annual reviews. Thus, together with the systematic approach to participant selection and follow-up, the POP study offers a more rigorous observational study dataset than many convenience samples. For the first time, the intensive rehabilitation groups were recruited from two different centers and the patients in the control group were followed in specialized PD centers that were also utilizing a multidisciplinary care approach, although this was mostly limited to an outpatient setting.

As described in the methods, the intensive rehabilitation programs implemented at the IR centers included many separate elements, such as aerobic exercise, gait and balance training, stretching, resistance training, and physical, occupational, and speech therapy, and could be of a variable duration, usually from 2 to 4 weeks.

Further study will be necessary to understand the contribution of each component, the benefits of their interaction, and the dose–response characteristics of this approach. There has been speculation that synergy between interventions has delivered a greater effect than the sum of the effects of the individual interventions [18]—this is an important topic for future research. High levels of weekly exercise have been associated with improved longitudinal outcomes [1]. Also, the present findings support intensive exercise versus moderate exercise with rehabilitation for motor performance and HRQL benefits, although this result may be affected by selection bias and should be evaluated in an RCT. Intensive aerobic exercise has been shown to yield metabolic benefits and enhance skill formation in animal models, and conditions that are favorable to synaptic plasticity [26]. Our findings could be explained by previous evidence that aerobic exercise increases BDNF expression throughout the brain, promotes BDNF and TrkB interaction, and lowers the threshold for LTP induction [27,28]. Also aerobic exercise may reduce neuroinflammation [29] and oxidative stress [30] while activating neurotrophin-signaling pathways [31], angiogenesis [32], and neurogenesis [33]. Similar effects have been found in patients with PD following intensive rehabilitation, with increases in BDNF serum levels and BDNF-trkB activation in lymphocytes [34,35]. Finally, a positron emission tomography with [18F] a DA-D2/D3R receptor, showed that 8 weeks of treadmill exercise in the early stages pf PD was associated with an increase in DA-D2R binding within the dorsal striatum [36], likely decreasing the inappropriate inhibitory drive of the indirect pathway [37]. Future work should investigate the hypothesis that intensive rehabilitation, with vigorous, repetitive, and challenging goal-based practice and aerobic training may promote neuroplasticity in the striatal-thalamic-cortical-motor circuit, which is responsible for automatic motor behavior. Other complementary rehabilitative approaches, including Tai Chi, active theatre, and dance may improve functional outcomes in patients with PD. In particular, both Tai Chi [38,39] and dance [40,41] seem to reduce motor symptoms, whereas active theatre [42] can be very effective for non-motor emotional aspects. According to a recent review, Tai Chi has beneficial effects on balance, walking ability, and gait velocity, but not on endurance and walking cadence [39]. On the other hand, dance can improve balance with small effect son freezing of gait. Future work should investigate the specific effects of the add-on use of these complementary therapies to intensive protocols.

Finally, our finding that the disease stage is always a significant covariate suggests that intensive rehabilitation may be particularly effective in the earlier disease stages. This is not only because intensive rehabilitation may be a promoter of neuroplasticity, but mostly because it may serve as a motivation and an *educational* or training period for maintaining a higher level of exercise when patients go back home.

In summary, the results of this study suggest that a regularly repeated program of intensive rehabilitation, interspersed with maintenance through a program of at least twelve hours of weekly exercise, including at least four hours of moderate to vigorous intensity, would be highly beneficial, not only to maintain or possibly improve motor performance and for the well-being of patients with PD, but also to reduce the caregiver strain.

Nevertheless, our findings must be interpreted with some degree of caution for several reasons. First among them, non-linearities in the disease severity versus mobility relationship may be a factor in these results, as the IR center cohort did have significantly more patients in the advanced stages of the disease compared to the control group. In addition, the linear adjustment model may fail to capture a divergence in the disease stage to the motor performance relationship in the extreme values of each. Indeed, this patient selection may bias the results and should be evaluated in an RCT study. Second, in this analysis we did not control for comorbidities, a factor that could especially influence the results of a lower rate of hospitalization for the patients in the IR centers. Third, the possibility to carefully review medication, the increased attention to the patients’ performance and needs, and the possibility to assess the patients for long period of time in the IR centers may play an important role in the motor and HRQL improvements. Fourth, the addition of another subgroup with intermediate levels of exercise without rehabilitation could have provided more strength to our conclusions. However, the number of patients for this subgroup was very small and could not be addressed in this study. Finally, this study does not allow an understanding of the contribution of each component of the intensive rehabilitation, the benefits of their interaction, and the dose–response effects.

While future investigation should address each of these confounding factors, the present results should provide the basis to prioritize further studies based on comprehensive community-based recruitment, to capture a cross-section of all individuals with PD, and to understand the effect of referral to expert centers on the overall burden of disease.

## 5. Conclusions

Despite the weaknesses of this study outlined in the previous paragraph, the results of this analysis, which is built on prior RCT evidence, suggest that this care model does deliver improved outcomes and can be replicated. Based on these findings, it would be valuable to continue to research the efficacy and reproducibility of evidence-based intensive rehabilitation strategies, with intervention, outcome, and, hopefully, biomarkers’ tracking to ensure continuous quality improvement.

## Figures and Tables

**Table 1:** The baseline characteristics of the two treatment groups: IR (intensive rehabilitation) and TO (traditional outpatient) centers.

	IR Centers (374)	TO Centers (387)	* *p*	N
**Sex:** * % female [N]*	40% [150]	40% [154]	0.989	40% [304]
**Age:** *median [range]*	69 [39–92]	70 [34–89]	0.329	758
**H–Y stages**			**<2.2 × 10^−16^**	761
H–Y stages *1–2* N	114	262		376
H–Y stage *3* N	196	106		302
H–Y stage *4* N	64	19		83
**Dx certainty:** % *>= 90% [N]*	79% [294]	82% [317]	0.292	80% [611]
**LED:** *median [range]*	696 [0–11,375]	500 [0–21,441]	**0.011**	761
**PD duration:** *median [range]*	9 [0–31]	5 [0–29]	**2.5 × 10^−16^**	758
**TUG score:** *median [range]*	11 [6–32]	13 [6–35]	**2.21 × 10^−4^**	665
**Falls:** *% reporting 1 or more [N]*	40% [151]	30% [117]	**0.004**	35% [268]
**MCSI:** *median [range]*	17 [0–52]	15 [0–57]	0.345	459
**PDQ-39:** *median [range]*	24.6 (0.8–96.1)	21.5 (0–82.4)	**0.009**	745
**Hospital adm:** % reporting *1+ [N]*	18% [66]	21% [81]	0.326	19% [147]

The differences between the two groups were tested with *t*-tests for continuous variables and with the chi-square test for categorical variables. TUG scores with penalty; PD duration as the years from diagnosis; MCSI was not available for all subjects as not all the subjects had caregivers. At the baseline, there were 3, 112, 302, 16, and 4 patients with missing data on PD duration, TUG penalty score, MCSI, PDQ-39, and hospital administration, respectively. Please notice that 80% of patients carried a diagnosis of idiopathic PD with 80% of certainty. Significant results (* *p* < 0.05) are reported in bold.

**Table 2:** TUG without push off: results of generalized linear mixed model.

Variable	Beta	se	t Value	*p*
(Intercept)	−3.288	1.094	−3.005	0.003
Follow up years	**0.290**	**0.117**	**2.478**	**0.013**
Sex = Male	−0.367	0.214	−1.716	0.086
Age at the baseline	**0.136**	**0.012**	**11.354**	**<2.00 × 10^−16^**
LED at the baseline	0.000	0.000	−0.466	0.641
H–Y stage = 3	**1.256**	**0.271**	**4.636**	**3.55 × 10^−6^**
H–Y stage = 4	**5.420**	**0.557**	**9.723**	**<2.00 × 10^−16^**
PD duration at baseline	0.001	0.023	0.046	0.963
TO centers group	**2.496**	**0.264**	**9.454**	**<2.00 × 10^−16^**
S2. Medium exercise and rehab subgroup	**1.423**	**0.488**	**2.914**	**0.004**
S3. No exercise subgroup	**2.341**	**0.467**	**5.015**	**5.31 × 10^−7^**

The results of the skew-T generalized linear mixed model with covariates at the baseline that best fitted the data for the TUG measures without push off. The number of subjects: 634, the number of visits: 1318. The TUG scores were set as missing if patients completed the test with push off or using an assistive device. Significant results are reported in bold.

**Table 3:** TUG with push off: results of the generalized linear mixed model.

Variable	Beta	se	t Value	*p*
(Intercept)	−2.886	1.231	−2.345	0.019
Follow up years	**0.645**	**0.128**	**5.734**	**9.79 × 10^−9^**
Sex = Male	−0.335	0.219	−1.168	0.243
Age at the baseline	**0.142**	**0.013**	**10.615**	**<2.00 × 10^−16^**
LED at the baseline	0.000	0.000	−0.246	0.806
H–Y stage = 3	**1.812**	**0.280**	**6.286**	**3.27 × 10^−10^**
H–Y stage = 4	**8.409**	**0.550**	**14.622**	**<2.00 × 10^−16^**
PD duration at baseline	−0.014	0.023	−1.138	0.255
TO centers group	**2.619**	**0.279**	**8.626**	**<2.00 × 10^−16^**
S2. Medium exercise and rehab subgroup	**1.308**	**0.577**	**2.140**	**0.032**
S3. No exercise subgroup	**2.236**	**0.559**	**4.012**	**6.01 × 10^−5^**

The results of the skew-T generalized linear mixed model with covariates at the baseline that best fitted the data for the TUG measures with push off. The number of subjects: 695, the number of visits: 1467. Significant results are reported in bold.

**Table 4:** The frequency of falls as binary variable (0 = “no falls”; 1 = “one or more falls”): the results of the generalized linear mixed model.

Variable	Beta	se	t Value	*p*
(Intercept)	−8.587	2.752	−3.120	1.80 × 10^−3^
Follow up years	−0.046	0.238	−0.190	0.846
Sex = Male	**−0.717**	**0.362**	**−1.980**	**0.048**
Age at the baseline	0.036	0.019	1.840	0.066
LED at the baseline	**0.001**	**0.001**	**2.420**	**0.016**
H–Y stage = 3	**2.189**	**0.879**	**2.490**	**0.013**
H–Y stage = 4	**2.619**	**1.087**	**2.410**	**0.016**
PD duration at the baseline	**0.142**	**0.049**	**2.910**	**0.004**
TO centers group	0.645	0.348	1.850	0.064
S2. Medium exercise and rehab subgroup	1.063	0.723	1.470	0.141
S3. No exercise subgroup	1.061	0.695	1.53	0.127

Results of the skew-T generalized linear mixed model with covariates at the baseline that best fitted the data for falls treated as a binary variable (0 = “no falls”; 1 = “one or more falls”). Number of subjects: 749, number of visits: 1803. Significant results are reported in bold.

**Table 5:** Hospital admissions as a binary variable (0 = “no”; 1 = “one or more”): the results of generalized linear mixed model.

Variable	Beta	se	t Value	*p*
(Intercept)	−3.030	0.670	−4.530	6.00 × 10^−6^
Follow up years	−0.038	0.097	−0.400	0.691
Sex = Male	0.137	0.140	0.980	0.329
Age at the baseline	−0.004	0.008	−0.480	0.629
LED at the baseline	0.000	0.000	1.570	0.117
H–Y stage = 3	**0.576**	**0.166**	**3.470**	**0.001**
H–Y stage = 4	**0.584**	**0.249**	**2.350**	**0.019**
PD duration at the baseline	**0.029**	**0.013**	**2.260**	**0.024**
TO centers group	**0.491**	**0.152**	**3.220**	**0.001**
S2. Medium exercise and rehab subgroup	0.460	0.352	1.310	0.190
S3. No exercise subgroup	0.365	0.340	1.070	0.284

Results of the skew-T generalized linear mixed model with covariates at the baseline that best fitted the data for hospital admissions treated as a binary variable (0 = “no”; 1 = “one or more”). The number of subjects: 755, the number of visits: 1762. Significant results are reported in bold.

**Table 6:** PDQ39: the results of the generalized linear mixed model.

Variable	Beta	se	t Value	*p*
(Intercept)	7.331	3.928	1.866	0.062
Follow up years	**1.621**	**0.260**	**6.238**	**4.42 × 10^−10^**
Sex = Male	**−5.955**	**0.774**	**−7.689**	**1.49 × 10^−14^**
Age at the baseline	**0.103**	**0.044**	**2.355**	**0.019**
LED at the baseline	**0.001**	**0.000**	**3.312**	**9.26 × 10^−4^**
H–Y stage = 3	**6.335**	**0.923**	**6.866**	**6.58 × 10^−12^**
H–Y stage = 4	**10.933**	**1.347**	**8.114**	**<2.00 × 10^−16^**
PD duration at the baseline	**0.483**	**0.076**	**6.371**	**1.87 × 10^−10^**
TO centers group	**1.946**	**0.825**	**2.359**	**0.018**
S2. Medium exercise and rehab subgroup	**5.027**	**2.128**	**2.362**	**0.018**
S3. No exercise subgroup	3.409	2.067	1.649	0.099

The results of the skew-T generalized linear mixed model with covariates at the baseline that best fitted the data for PDQ39, an index of health-related quality of life. The number of subjects: 753, the number of visits: 1732. Significant results are reported in bold.

**Table 7:** MCSI: the results of the generalized linear mixed model.

Variable	Beta	se	t Value	*p*
(Intercept)	1.832	3.755	0.488	0.626
Follow up years	**0.972**	**0.279**	**3.484**	**4.94 × 10^−4^**
Sex = Male	1.276	0.839	1.521	0.128
Age at the baseline	0.018	0.043	0.415	0.678
LED at the baseline	0.001	0.001	1.039	0.299
H–Y stage = 3	**5.549**	**0.906**	**6.123**	**9.19 × 10^−10^**
H–Y stage = 4	**11.183**	**1.485**	**7.531**	**5.03 × 10^−14^**
PD duration at the baseline	**0.246**	**0.074**	**3.339**	**0.001**
TO centers group	**1.966**	**0.837**	**2.348**	**0.019**
S2. Medium exercise and rehab subgroup	**5.046**	**1.901**	**2.655**	**0.008**
S3. No exercise subgroup	**4.085**	**1.797**	**2.274**	**0.023**

The results of the skew-T generalized linear mixed model with covariates at the baseline that best fitted the data for MCSI, an index of caregivers’ strain. The number of subjects: 499, the number of visits: 1023. Significant results are reported in bold.

## Data Availability

Data are available upon request to the Parkinson’s Foundation.
